# Systematic development of CHEMO-SUPPORT, a nursing intervention to support adult patients with cancer in dealing with chemotherapy-related symptoms at home

**DOI:** 10.1186/s12912-018-0297-8

**Published:** 2018-06-27

**Authors:** Annemarie Coolbrandt, Hans Wildiers, Bert Aertgeerts, Bernadette Dierckx de Casterlé, Theo van Achterberg, Koen Milisen

**Affiliations:** 1 Department of Oncology Nursing, University Hospitals Leuven, Herestraat 49, 3000 Leuven, Belgium; 2 Department of Public Health and Primary Care, Academic Centre for Nursing and Midwifery, KU Leuven, Leuven, Belgium; 3 Department of General Medical Oncology, University Hospitals Leuven, Leuven, Belgium; 4 Department of Public Health and Primary Care, Academic Centre for General Practice, KU Leuven, Leuven, Belgium

**Keywords:** Chemotherapy, Symptoms, Nursing, Intervention, Complex intervention, Intervention-mapping approach, Self-management

## Abstract

**Background:**

Given the great symptom burden associated with chemotherapy on the one hand and generally poor self-management of symptoms by cancer patients on the other hand, our aim was to develop a nursing intervention to reduce symptom burden in adult cancer patients treated with chemotherapy and to support them in dealing with their various symptoms at home.

**Methods:**

Development of the intervention was guided by the Intervention Mapping Approach and included following steps: needs assessment, formulation of proximal programme objectives, selection of methods and strategies, production of programme components, and planning for implementation and evaluation of the intervention. A panel of multidisciplinary healthcare professionals (*n* = 12) and a panel of patients and family caregivers (*n* = 7) were actively involved developing the intervention at each stage.

**Results:**

For the intervention, four patient performance objectives relating to self-management were advanced. Self-efficacy and outcome expectations were selected as key determinants of dealing with chemotherapy-related symptoms. As methods for supporting patients, motivational interviewing and tailoring were found to fit best with the change objectives and determinants. Existing patient information materials were re-designed after panel input to reinforce the new intervention approach.

**Conclusion:**

The intervention mapping approach, including active involvement of the intervention providers and receivers, informed the design of this nursing intervention with two or more contacts. Further evaluation is needed to gain insight into the potential effects, feasibility and mechanisms of this complex intervention.

**Electronic supplementary material:**

The online version of this article (10.1186/s12912-018-0297-8) contains supplementary material, which is available to authorized users.

## Background

Chemotherapy is associated with multiple, often distressing, side effects. The negative impact of these on quality of life is widely recognized [1, 2]. Typically, these side effects are experienced at home, in the absence of professional assistance [3]. Consequently, chemotherapy that includes ambulatory treatments forces patients to actively self-manage their symptoms. However, few patients seem to be able to do so adequately [4]. Performance of symptom self-management strategies is generally poor [5–8]. Also, patients sub-optimally report their symptoms to healthcare professionals [9, 10]. Patients report lacking knowledge and experience [11], and report high levels of unmet needs in relation to self-care support [12]. Evidence suggests that greater symptom burden is associated with poorer self-care [8, 13].

The burden of chemotherapy-related symptoms and (often unmet) patient needs related to their self-management has catalysed the development of several new nursing interventions to address these issues [12, 14–16]. Many have focused on managing a single symptom, such as oral mucositis or fatigue, but it is likely that meaningful improvement in quality of life can only be achieved by interventions that focus on multiple symptoms that cancer patients face [17]. Some interventions targeting multiple symptoms have indeed produced a positive impact on symptom burden [18, 19]. However, a recent systematic review revealed that these interventions have produced inconsistent results [20]. Combined with variable degrees of efficacy, many of these intervention studies face reproducibility limitations. Some studies contain little description of the studied interventions, their core components and intervention development [17, 21, 22]. The usual care that was employed for comparison is generally poorly described [20]. Intermediate outcomes, contributing to a better understanding of the effect mechanism of the intervention, are evaluated and reported in only one study [23]. Also, qualitative data on the intervention is presented in only one case [24–27]. Consequently, many questions remain unanswered: How were outcomes reached? Which intervention components produced measurable effects and by what mechanism(s)? Also, what factors promoted or hindered their results? [28, 29].

While the systematic development of complex interventions using the best available evidence and appropriate theory is becoming increasingly encouraged and acknowledged [30–32], such approaches are rarely applied or reported in interventions targeting chemotherapy-related symptom burden [20]. The Intervention-Mapping Approach (IM) is a conceptual framework for systematically developing healthcare programmes [33–35]. It has been used to further advance theory and evidence-based health promotion programmes in many health domains, such as smoking cessation, preventing HIV transmission, sun protection, asthma management, etc. [34] The framework assists programme developers in making and documenting decisions for influencing change in behaviour and improving health, while making use of available evidence and theory and collaborating with future intervention providers and receivers. Using IM in intervention development is presumed to improve the potential effects of healthcare programmes [34].

This paper describes a step-by-step overview of the development of a nursing intervention aimed at reducing chemotherapy-related symptom burden. We call it CHEMO-SUPPORT. In the development process, we used the best available evidence and theory and employed the IM Approach. The second aim of this paper is to fully describe the actual intervention, as it will be implemented and studied. We used the Template for Intervention Description and Replication (TIDieR) checklist to describe our intervention. The TIDieR is presented as an extension of the CONSORT 2010 statement and the SPIRIT 2013 statement with the aim of improving the completeness of reporting and ultimately the replicability of interventions [36].

## Methods

We followed the Intervention Mapping Approach of Bartholomew et al. [34] to guide development of the intervention. Table 1 summarizes the 6 steps and their specific objectives. It also provides an overview of the methods used and the results obtained at each step.

**Table 1:** Overview of the step-by-step process of Intervention Mapping with methods used and results produced at each step

	Methods	Results
Step 1 Needs assessment: Objectives: establishing participatory planning groups, conducting the needs assessment, specifying desired programme goals	♦ Literature review of symptom self-management in patients with cancer ♦ Qualitative study on dealing with chemotherapy-related symptoms at home [3] ♦ One discussion session with professional panel and one with patients and caregivers panel	♦ Needs structured in PRECEDE-model (Fig. 1) ♦ Reported and observed behavioral problems: patients’ poor/inadequate self-management, poor communication and reporting of chemotherapy-related symptoms ♦ Desired program goals: improving self-management and communication/reporting of chemotherapy-related symptoms
Step 2 Matrices of proximal programme objectives Objectives: stating behavioral and environmental outcomes of the intervention, defining clear performance objectives (POs), creating matrices of change objectives by crossing POs with determinants	♦ Outline of matrices of proximal programme objectives by project leader ♦ Review of theory and outline of potential determinants ♦ One discussion session with professional panel and two discussion sessions with patients and caregivers panel	♦ Consensus on four patient performance objectives (POs): * Preventing, monitoring, reporting and managing chemotherapy-related symptoms at home* ♦ Consensus reached on vital determinants: * Self-efficacy and outcome expectations of patients* ♦ Matrices of proximal programme objectives for future program receivers (patients) (example in Table 2) ♦ Definition of nursing objectives to support patients’ performance objectives
Step 3 Selecting theoretical methods and practical strategies Objectives: generating programme ideas, identifying and selecting theoretical methods, selecting or designing practical applications	♦ Study of methods and theories [16, 18, 19] ♦ Evaluation of the ideas on methods and strategies yielded in the earlier panel meetings ♦ Systematic review of complex nursing interventions aimed at reducing chemotherapy-related symptom burden [10] ♦ One discussion session with professional panel and one with patients and caregivers panel ♦ One discussion session with nursing panel	♦ Consensus on principal methods of the intervention: tailoring and motivational interviewing ♦ General outline of the intervention (Fig. 2): Brief motivational intervention, advanced on the basis of estimated individual need ♦ Formulation of additional project objective for the purpose of the intervention: revision of written patient information and advices
Step 4 Producing programme components Objectives: determining preferences for programme design, creating programme scope and sequence, preparing design, reviewing, developing and pretesting programme materials	Intervention manual development: ♦ Formulating nursing approach at every patient contact in the program ♦ Discussion with project team, nursing panel, 2 onco-psychologists	Final intervention manual produced
	New written patient information development: ♦ Web survey eliciting patient feedback (*n* = 102, characteristics see Table 3) on information and advice for 19 chemotherapy-related symptoms (question format see Table 1) ♦ First revision and second patient feedback round (*n* = 21) ♦ Feedback and discussion with healthcare professionals (*n* = 17)	New booklet produced “Dealing with side effects from chemotherapy at home”, outlining the 4 recommended self-management behaviours and presenting information and (professional and fellow patient) advice on 19 side effects
Step 5 Planning for adoption, implementation and sustainability Objectives: identifying potential adopters, stating outcomes for programme use, specifying determinants and creating matrices (defining determinants and change objectives) for programme adoption, implementation and sustainability	Planning for the implementation of the intervention in an intervention study: ♦ Planning selection strategy and criteria for the intervention providers in the study ♦ Translating nurse POs into training programme for the intervention nurses ♦ Outlining communication strategy for clinical nurses and other healthcare professionals	♦ Selection of 6 intervention nurses ♦ 2-day long training programme for the intervention nurses ♦ Meetings (*n* = 9) with clinical nurses (*n* = 114) ♦ Meetings with doctors and paramedics
Step 6 Planning for intervention evaluation Objectives: describing programme outcomes, writing evaluation questions, developing indicators and measues, specifying evaluation design	Together with the project team: ♦ Translating health and quality of life targets, POs and determinants into study outcomes ♦ Choosing appropriate methods and study design	♦ Protocol of a mixed-methods study ♦ Qualitative approach to explore patient experience with the intervention: satisfaction with intervention, open questions and semi-structured interviews ♦ Quantitative approach to study intervention effect: experimental before-after study with sequential design * Primary outcome: Symptom distress* * Secondary outcomes:* * Symptom severity* * Self-efficacy* * Outcome expectations* * Self-care*

Two groups of individuals were involved throughout development of the intervention. A panel of multidisciplinary health professionals from several different centres comprised one group. This panel included 3 oncologists and 4 nurses from 3 different hospitals (1 academic and 2 non-academic); 1 general practitioner; 3 home care nurses from different primary care organisations; and 1 psychologist, with expertise in self-management of chronic disease.

The other panel of individuals included patients and caregivers. Five of them were patients who had been treated with chemotherapy, and 2 were family caregivers (spouses in both cases). They represented patients/caregivers from 3 different hospitals (1 academic and 2 non-academic). Patients were recruited with the help of nurses and doctors from the hospitals, or through self-help groups. One participating caregiver also came from a self-help group, other caregivers from a group session for partners of people with cancer.

The cancer diagnoses associated with the seven participants comprising the patient and caregiver group spanned a mix of diagnoses (haematological cancer, digestive tract cancer, breast cancer, brain tumour, and gynaecological cancer). Three patients were women and 2 were men, 1 caregiver was a man, and the remaining one was a woman. The mean age in this panel was 54 years. Patients’ age ranged from 18 to 69 years. This mix was important to achieve a diversity of perspectives [34] and to produce an intervention that is employable and generalizable to patients with cancer regardless of their demographical or clinical variables.

All panel members participated in five meetings for which they were compensated. Anonymity, confidentiality, non-binding and well-informed participation were closely guarded as ethical principles of the panel members’ involvement.

Every panel meeting had its specific objectives according to the stage in the IM process, e.g. validating the needs assessment and getting consensus about the program objective. Relevant evidence was collected in preparation for panel meetings and additional literature was searched after collaborative consultation at the meetings, when necessary. The project leader (A.C.) applied different techniques to facilitate interpersonal communication, idea generation and consensus: e.g. responding to a paper or presentation of evidence, brainstorming, nominal group technique.

### Step 1: Needs assessment

The needs assessment component comprised a qualitative study of how adult chemotherapy patients deal with side effects at home [13], a literature review of how cancer patients manage their symptoms, the development of a needs assessment model and an independent discussion with each panel. Panel discussions were conducted in order to discuss the model and to gain insights into the relative importance of behavioural and environmental factors and their determinants.

### Step 2: Matrices of proximal programme objectives

Two independent meetings with the panels formulated the most relevant behavioural outcomes and necessary performance objectives (POs). The latter described what intervention receivers and performers “need to do in order to accomplish improvement in health outcomes” ([16], p. 239). These meetings also yielded a preliminary set of determinants. An additional meeting was held with the intended programme recipients (i.e., the patient and caregiver panel) to further discuss and prioritise the determinants for each PO.

### Step 3: Selecting methods and strategies

In selecting theoretical methods and practical strategies appropriate for the intervention, the panels took into account the evidence on methods and theories linked with the change objectives and determinants recommended by the panel members [34, 37, 38], methods and strategies used by other nursing interventions aimed at reducing chemotherapy-related symptom burden [20] and methods and strategies suggested by the panel members during previous patient/caregiver and professional meetings. A provisional draft of the intervention was discussed with both panels to further refine the new nursing intervention. An auxiliary nursing panel was organised to query oncology nurses (the future intervention providers) for their opinions on the perceived relevance and feasibility of the intervention.

### Step 4: Producing programme components

The first component that needed to be developed was a detailed scenario or plan of action for executing the nursing intervention. We call this the intervention manual. The intervention manual described every relevant patient contact, from start of the intervention to programme termination.

A second objective prioritized at this stage was to reorient the currently used patient information tools to fit the new intervention. Some members of the patient/caregiver panel proposed that this was necessary so that patient information could better support and empower the determinants of the programme receivers’ POs, i.e., self-efficacy and outcome expectations. An online survey was set-up to obtain patient testimonies and feedback. Its aim was to produce improved phrasing and to complete the symptom description and self-care advice in order to better reflect the patients’ perspective and experience. Additionally, quotes that well supported, illustrated, or supplemented the professional advice were extracted. The survey overview is illustrated in Table 2. Patients’ online feedback was anonymous and confidential, and was not reported in any other form than its contribution to the re-writing of our patient brochure, to which they consented as part of the online participation. The web survey was advertised by hanging posters and flyers in the different oncology wards, by notifying self-help groups, and by posting content on the hospital’s website and the website of ‘Kom op tegen Kanker’ (i.e., a cancer care and research charity in the local context). Patients, as well as healthcare professionals, provided additional oral or written feedback, as the patient information was re-written in subsequent versions.

**Table 2:** Overview of survey used to reorient currently available patient information with proposed information for new intervention

	Topic	Question	Answer
Question 1	Symptom experience	What would you want to delete, adjust, add to the current patient information on (this side effect)? What would you want to tell fellow patients about (this side effect)?	Freely able to answer
Question 2*a	Self-care advice	To what extent is this advice helpful for (this side effect)?	4-point Likert scale
Question 2*b	Self-care advice	Why or why not is/was this advice helpful to you? What would you want to share with fellow patients about this advice?	Freely able to answer
Question 3	Self-care advice	Which other advices or strategies have helped you to deal with (this side-effect)? Which other advices would you share with fellow patients?	Freely able to answer
Question 4	Social support	How can your social network play a part in dealing with (this side effect)?	Freely able to answer
Question 5	Other	Which other suggestions do you have for patient information on (this side effect)?	Freely able to answer

*Repeated as many times as there was advice on the particular side effect

### Step 5: Planning for adoption, implementation and sustainability

Our primary intention for the intervention at this point was to conduct a pilot study instead of have the intervention immediately adopted in daily care.

To ensure treatment fidelity during the pilot study, we chose not to involve all clinical oncology nurses as possible programme providers but instead to have a limited group of trained intervention nurses conduct the intervention. Therefore, objectives for Step 5 were selecting the intervention nurses, planning their training programme, and coordinating and integrating the intervention with the usual care that would be delivered by the clinical nurses and doctors.

Next, a consultation and information plan was set up to present the project and to address possible concerns of clinical nurses, doctors, and paramedics who would be involved in the care for patients participating in the study.

### Step 6: Planning for evaluation

The final step of the intervention development comprised the preparation of the evaluation of the intervention. A protocol for a mixed-method pilot study was written in order to capture the intervention effects and, at the same time, grasp the recipients’ responses to the intervention and to explore explanations of the quantitative findings [39].

## Results

In the results section, we present the outcomes and decisions made at each step of the intervention development process. Evidence and panel opinions supporting these decisions are available in Additional file 1.

### Step 1: Needs assessment

**Fig. 1:**
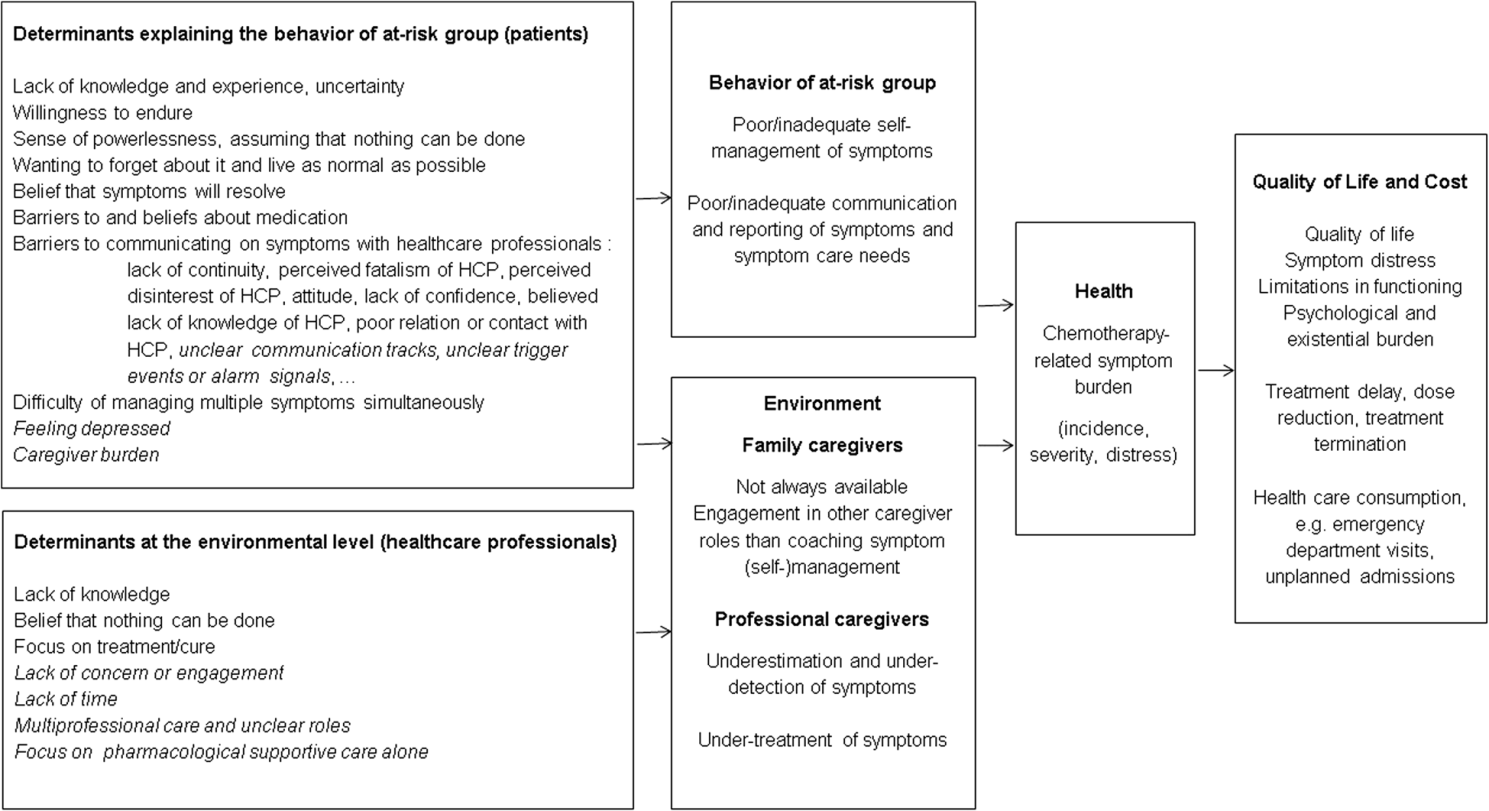
Needs assessment. Items proposed by the panels are italicized. The remaining items are from the literature

The results of the needs assessment are presented in Fig. 1. The evidence underpinning the needs assessment is available in Additional file 2. The complete results of our qualitative study into how adult patients receiving chemotherapy deal with treatment-related symptoms at home is reported elsewhere [13]. Both panels agreed that coaching patients to self-manage symptoms adequately was the appropriate goal for the intervention.

### Step 2: Matrices of proximal programme objectives

Based on the needs assessment, the panels agreed on four patient performance objectives (POs) for the self-management intervention:

1. PO1: The patient performs preventive self-care behaviour, addressing the possible side effects related to his/her chemotherapy treatment.
2. PO2: The patient monitors the severity and duration of his/her symptoms.
3. PO3: The patient adequately reports in a timely manner and discusses his/her symptoms with healthcare professionals.
4. PO4: The patient performs self-care behaviour to manage symptoms.

Professionals and patients selected *self-efficacy*, *outcome expectations*, *knowledge* and *social support* as vital determinants for these POs. Interestingly, patients and caregivers suggested that tackling outcome expectations, self-efficacy, and social support would be especially able to increase the potential effectiveness of the intervention, as they believed the need for knowledge was already largely addressed in standard care. The professional panel shared this opinion on priorities.

Performance objectives and determinants were crossed in matrices to arrive at clear proximal programme objectives. An example for PO1 is provided in Table 3. To target the environmental factors and mainly the nursing role, the patient POs were translated into nursing POs.

**Table 3:** Examples from the matrix of proximal programme objectives for patients and nurses for PO1

PO1: The patient performs preventive self-care behaviour related to possible side effects of chemotherapy treatment.				
Determinant	Knowledge	Outcome expectations	Self-efficacy	Social support
Patient	Patient describes necessary self-care measures to prevent possible side effects from treatment. For example, finding balance between rest and exercise/activity to prevent fatigue	Patient expresses conviction that self-care measures will help to prevent side effects or to prevent side effect from getting severe.	Patient expresses confidence in their capability of performing the relevant self-care measures.	Patient involves his family caregivers to remind and support him in performing preventive self-care behaviour.
Nurse	Nurse instructs patient on relevant self-care measures to prevent possible side effects form his treatment.	Nurse explains effects and preventive mechanisms of preventive self-care behaviour. For example, importance of physical activity in maintaining physical condition and preventing fatigue from worsening	Nurse queries patients on perceived barriers for performing the self-care measures.	Nurse explores possible social support for reminding and supporting the patient with preventive self-care at home.

### Step 3: Selecting methods and strategies

**Fig. 2:**
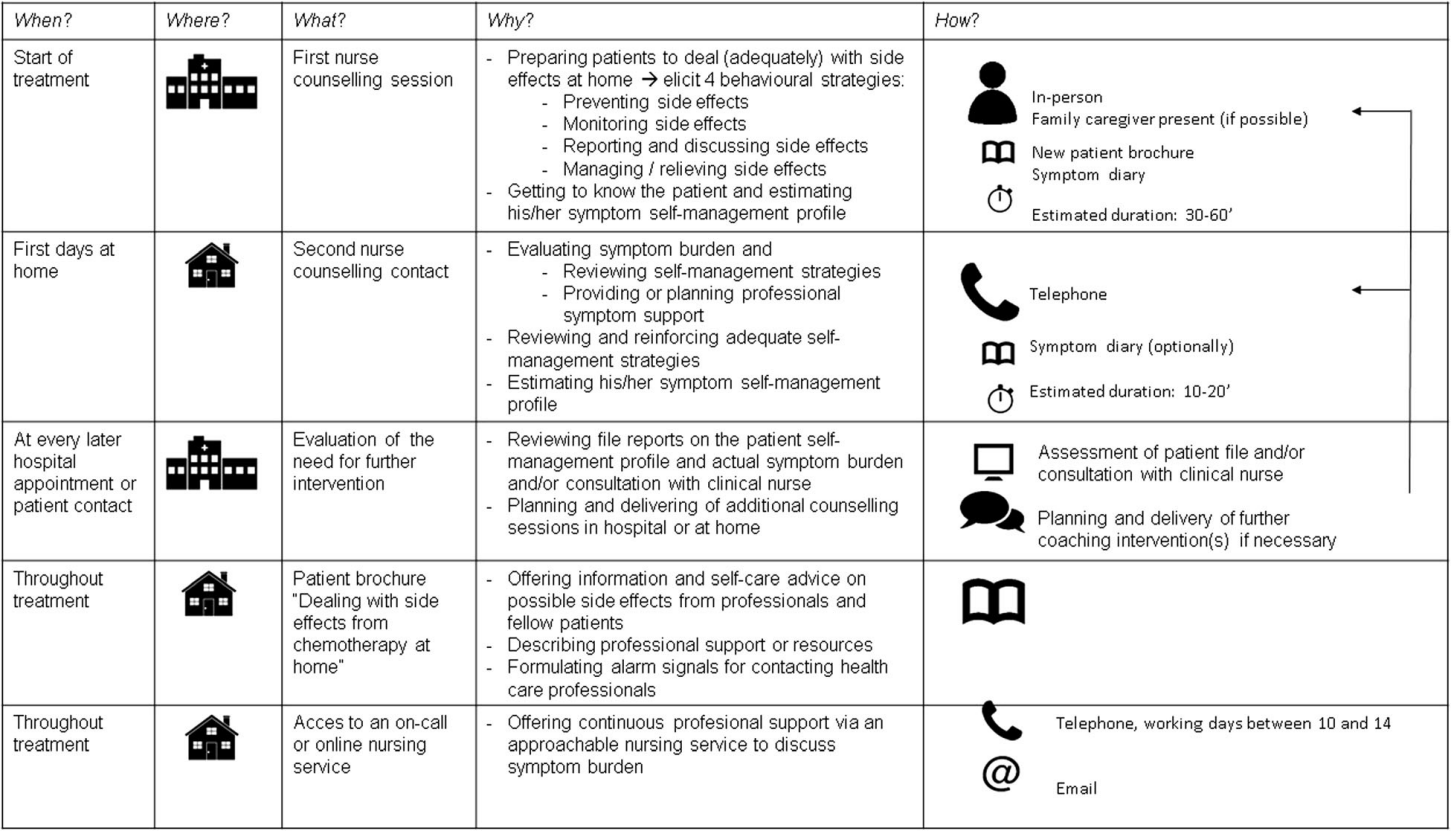
Intervention overview

Both panels agreed that tailoring was an important strategy to increase the intervention’s potential efficacy. Naturally, this involved tailoring the intervention content to the particular treatment being started (and the possible side-effects associated with that treatment). It also meant taking into consideration the patients’ personal symptom experience and symptom-management style [13]. More importantly, however, both panels agreed on the need to tailor the intervention dose. A standard intervention dose of two sessions was considered sufficient and feasible for patients who, with the help of the intervention, expressed sufficient knowledge, motivation and social support to perform the behavioural objectives. More sessions seemed warranted for those patients who were more at risk (e.g., living alone or poor social support, poor understanding of information). Figure 2 presents an overview of the intervention.

Based on the panel meetings and the relevant determinants, motivational interviewing was believed to be the crucial foundation for the coaching intervention. Motivational interviewing (MI) finds its origin in the Transtheoretical Model, which presumes people are in different stages of readiness to make behavioural changes. It is a goal-directed counselling style for eliciting behavioural change, holding to the principle that motivation is elicited from the patient and not imposed from outside [40–42]. As a counselling style, MI itself encompasses other methods such as reinforcement and self-reevaluation that is applied in the intervention to elicit behavioural change.

### Step 4: Producing programme components

For the intervention providers, a plan of action was developed that detailed the behaviour of nurses at every patient contact. Both motivational interviewing and tailoring were explicitly included and were outlined in the manual. Communication and motivational techniques were illustrated with examples of phrasings. The complete intervention manual is available upon request to interested readers.

One hundred-two patients between 27 and 78 years old, most of them (69%) women, participated in the online survey. Twenty-one survey respondents provided further feedback on rewritten information by email or during a personal meeting. Also, 17 healthcare professionals (psychologists, sexologists, dieticians, a physiotherapist, a revalidation therapist, and nurses and doctors) provided feedback on side effects related to their clinical expertise. The new booklet is called, “Dealing with side effects from chemotherapy at home”.

### Step 5: Planning for adoption, implementation and sustainability

We used the following criteria to recruit and select the intervention nurses that would deliver the intervention during the pilot study:

1. Having a bachelor’s degree in nursing.
2. Having clinical oncology nursing experience. Having an extra degree in oncology nursing was considered valuable but not necessary.

Given the anticipated caseload of 2.3 new patients, or between 1 and 7 new patients per day, we sought 1.2 full-time equivalent positions so that one intervention nurse would always be available on every working day. We explicitly divided the mandate over six different nurses, since the allocation of one fixed nurse per patient was not the aim of the intervention and would potentially make the intervention more about the trust relationship between patient and nurse than about the intended active ingredients of the intervention. The six selected intervention nurses were all women, were between 37 and 50 years old, and had between 6 and 19 years of oncology nursing experience.

Nursing POs guided the content chosen for the training of the intervention nurses. A 2-day long training programme was organised to share knowledge and to provide training on the skills needed to meet the nursing POs. It included a thorough presentation of the intervention and the intervention manual, motivational interviewing, symptom management during chemotherapy, with a focus on self-care and (multidisciplinary) professional care, presentation of the new patient brochure, registration of patient care activities, and intervention fidelity.

Meetings were held to present the project to the ward nurses. The main purpose of these meetings was twofold: first, to ensure that clinical nurses would have sufficient knowledge about the intervention; and second, to engender in them a positive attitude towards the intervention and its integration into standard care. Next, the project aims and its content were discussed at meetings of the board of directors of all medical wards involved.

### Step 6: Planning for evaluation

The project team selected symptom distress as a primary outcome. We believed that it was sensitive to the components, and it matched the goals of the nursing intervention. Symptom severity and number of symptoms were selected as secondary health and quality-of-life outcomes. Next, the performance objectives and determinants suggested three intermediate outcomes for the study:

1. self-efficacy,
2. outcome expectations,
3. self-care or the adequacy of patient’s self-management behaviour.

These were seen as especially important for gaining insight into the intervention’s mechanism, or lack of an effect. Given the novelty of the intervention and given the recommendation of mixed methods research for the evaluation of complex interventions [43], the project team considered a qualitative evaluation of the patients’ experience with the nursing intervention equally important as the quantitative pilot study to explore its potential effects. Finally, it was decided to monitor intervention fidelity by having the intervention nurses report on the completeness of and adherence to the intervention components at each patient contact. After every patient encounter, intervention nurses self-rated the contact on the extent they believed they had addressed the core elements of the intervention. Protocol of this mixed-methods evaluation and its results are reported elsewhere [44].

## Discussion

We systematically developed a nursing intervention—called CHEMO-SUPPORT—aimed at reducing chemotherapy-related symptom burden that patients experience at home. This process resulted in an intervention including in-person coaching, telephone counselling, written patient information, and online/on-call access to nursing support. The intervention uses a tailored motivational approach instead of the educational approach for transferring standardized information and advice to the patient, unlike what is currently used in standard care.

Overall, earlier studies on nursing interventions targeting chemotherapy-related symptom burden have been unclear about intervention development methods [20]. Developing complex interventions using evidence and theory has been increasingly encouraged [30, 31], and by doing so, it is assumed that intervention developers improve the intervention’s potential effects. Consistent with this hypothesis, the clinical utility of using a systematic development approach for our intervention should be judged on the results of the intervention, which will be reported later. However, reporting of the intervention development process alone has merit in clarifying the chosen objectives, methods, and strategies of the intervention. This will help readers and other programme developers not only to adequately interpret intervention results but also to replicate or build on research findings and further adapt the intervention content and delivery modalities [36].

Designing interventions using IM is a time-consuming process. Yet, we believe the mandate of having a clear focus, objectives, methods, and strategies, alongside stepwise planning of the intervention and inclusion of evidence and professional and patient expertise, is crucial in shaping the intervention toward its final content. For example, the complete make-over of our standard patient information was not anticipated at the start of this study but resulted from the patient panel’s clear statement that reorientation of information material was necessary for better supporting patients in meeting their POs. As we moved through the six steps of IM, our decisions shaping the intervention were evidence- and theory-informed to the greatest extent possible. Meetings with the panels at each step of IM helped to complement the evidence with clinical and patient experience and to make clinically relevant and patient-centred decisions, all of which helped us to move forward in the development process.

It is important to note the limitations of our process of systematic intervention development and, as a consequence, of our intervention. First, social support as a determinant for adequate symptom self-management has received relatively little attention in the development of the intervention. While social support is clearly addressed in the counselling manual, the intervention could probably still benefit from better matching methods to change and mobilise social support [34, 40]. From what is known about the role of social support during treatment with chemotherapy, family caregivers’ role, as well as patients’ expectations, is very variable [45–48]. Caregivers sometimes act as co-managers of side effects, or sometimes as coaches. However, caregivers experience the patient’s disease, treatment, and symptoms differently than the patient. So some patients feel that this difference prevents spouses and family members from developing a partnership to deal with all the burden. Also, some patients don’t feel the need to have someone support their symptom management, while others simply have no one available. More in-depth research is needed to come to a better understanding of how to engage family caregivers in the symptom management process.

Secondly, our intervention development was mainly directed at tackling patient-related determinants of poor symptom self-management. Concerning the environmental-level determinants (see Fig. 1), lack of time and concern were addressed by hiring highly motivated auxiliary nurses who believed in the value of the intervention. Thus, the decision to plan an intervention study allowed us to delay dealing with some of the environmental determinants, specifically time and attitude. These issues will surface again as we discuss the adoption and sustainability of the intervention in daily practice. However, both quantitative and qualitative evidence on CHEMO-SUPPORT will facilitate the planning of further actions towards future programme providers and policymakers.

Finally, we did not pilot test the intervention or intervention components with intended programme receivers, as is recommended as part of step 4 of IM [34]. Given the active involvement of patients as well as professionals on the one hand, and our familiarity with the implementation of interventions in this clinical domain and in this clinical setting on the other hand, we were confident that the intervention could be delivered as planned. Also, we planned thorough qualitative evaluation of the intervention alongside our quasi-experimental study. However, pilot testing remains useful for getting a sense of the possible effects, determining how the intervention is perceived by naïve patients (i.e., those who have not participated in the intervention development process), and in determining problems with implementation [34]. Ultimately, our mixed-methods evaluation will guide the revision of the intervention before further implementation.

## Conclusion

We used the IM Approach to design a self-management intervention aimed at reducing chemotherapy-related symptom burden at home. Given the impact of chemotherapy-related symptoms and the outpatient organisation of cancer treatment, self-management is a logical goal for nursing care. However, generally poor self-management suggests that well-designed nursing interventions are imperative. The combination of evidence, theory, and clinical and patient experience in the step-by-step IM Approach resulted in a clearly described self-management support intervention to be tailored according to the patient’s self-management profile. The complete description of the intervention in this developmental study provides a foundation on which others can build on for future research and practice.

## Additional files

Additional file 1: File presents evidence and opinions supporting decision-making throughouth the Intervention Mapping stages, from needs assessment to final CHEMO-SUPPORT intervention. (DOCX 98 kb)

Additional file 2: File shows evidence underpinning the needs assessment. (DOCX 66 kb)

## Abbreviations

IM: Intervention mapping
MI: Motivational interviewing
PO: Performance objective
