# Supplementary material for: Systematic development of CHEMO-SUPPORT, a nursing intervention to support adult patients with cancer in dealing with chemotherapy-related symptoms at home
Source: BMC Nurs. 2018 Jun 27;17:28. doi: 10.1186/s12912-018-0297-8 (PMC6020323; doi:10.1186/s12912-018-0297-8)
Supplement: Supplementary file 1 — File presents evidence and opinions supporting decision-making throughouth the Intervention Mapping stages, from needs assessment to final CHEMO-SUPPORT intervention. (DOCX 98 kb) [file 12912_2018_297_MOESM1_ESM.docx]

**Additional file 1. Evidence and Opinions supporting Decision-Making from Needs Assessment to Final CHEMO-SUPPORT Intervention**

|  | **Outcome and elaboration** | **Evidence and opinions supporting decision-making** |
| --- | --- | --- |
| **Programme goal** | Coaching patients to adequately self-manage chemotherapy-related symptoms at home | - The programme goal was selected by both panels based upon the needs assessment (see Figure 1), mainly: the inadequate symptom self-management and reporting of symptoms.^1-6^ - That professional caregivers are currently providing suboptimal support to assist patients in dealing with symptoms at home -as supported by qualitative evidence^7-9^, the experiences within the patient and caregiver panel as well as from the professional panel- endorsed the relevance of the desired programme goal. - At the same time, both panels believed that –given the ambulatory setting of (more and more) cancer treatments- developing effective interventions for self-management support is highly important. - Finally, evidence that self-care behaviours can be learnt and improved by nursing interventions confirmed the selection of the programme goal.^2, 3, 10^ |
| **Performance objectives (PO)** | PO1: The patient performs preventive self-care behaviour.  PO2: The patient monitors the severity and duration of his/her symptoms.  PO3: The patient adequately reports in a timely manner and discusses his/her symptoms with healthcare professionals.  PO4: The patient performs self-care behaviour to manage symptoms. | - The panels selected and endorsed 4 performance objectives based upon the needs assessment (see Figure 1), the behavioral factors explaining or contributing to the health problem in particular. - PO1 and PO4 were supported by the evidence that patients’ self-management of cancer- or treatment-related symptoms is poor^1-4^. Patients perform few self-management strategies although higher number of self-management strategies is associated with better symptom response.^4^ - PO3 was supported by the evidence that patients poorly or inadequately report or communicate about symptoms with their health care professionals^5, 6^. - PO2 was supported by the evidence that professional caregivers underestimate symptom burden^11-13^ and that therefore, patient-reported outcomes are an essential basis for adequate symptom-management support. Also, delayed patient-reporting of symptoms and symptom severity (at the time of a hospital contact) results in underestimation of symptom burden.^5^ Thus, daily self-report is recommendable. Moreover, it improves patient-clinical communication.^14-16^ Finally, self-monitoring improves patients’ insight in the evolution of their symptoms and the effects of their symptom-management strategies, and therefore: it helps patients to perform adequate self-management.^17, 18^ |
| **Key determinants** | Self-efficacy and outcome expectations | - Evidence shows that patients feel powerless, out of control or fatalistic regarding to chemotherapy-related symptoms^7-9, 19^, and this negatively affects their self-management behaviour. Therefore, perceptions of control have been proposed as an important barrier and an essential target to encourage active involvement in self-care.^19^ - The relationship between an individual’s beliefs, his behaviour, and even his health outcomes is theoretically accepted.^20^ - This relation is supported by empirical evidence showing a statistically significant relationship between self-care agency and use of self-care measures: women who had higher scores before starting treatment used more self-care measures for chemotherapy side effects.^21^ - From their own experience, the patient and caregiver panel supported the importance of self-efficacy and outcomes expectations. |
| **Theoretical method** | Motivational interviewing and tailoring | - Our qualitative research demonstrated how a complex set of dynamic personal and environmental factors results in very *personal* symptom experiences and symptom management styles.^8^ Both panels agreed that an individual approach with respect to personal experience and motivation was therefore essential. - Based on Theories of self-regulation, motivational interviewing is a collaborative, goal-oriented style of communication aimed at strengthening personal motivation for and commitment to a specific goal by eliciting and exploring the person’s own reasons for change within an atmosphere of acceptance and compassion.^22, 23^ MI itself encompasses other methods such as reinforcement and self-reevaluation that are relevant methods for behavioural change. - Tailoring, i.e. matching the intervention or components to previously measured characteristics of the participant, is proposed as a basic method for health promotion programs at the individual level.^22, 23^ - Evidence suggests that education, as was the standard of care in our setting, positively influences knowledge but doesn’t produce behavior change and that other methods are needed for behavior change. At the same time, motivational interviewing has been proved to produce behavior change.^24-26^ |
| **Intervention dose** | Low standard dose of two contacts, to be tailored and with the option of escalation for patients at risk for and/or showing inadequate self-management | - Published nursing interventions aimed at reducing chemotherapy-related burden apply highly variable intervention dose and intensity^27^. Interventions with a fixed number of contacts used 3 to 10 sessions. One of the more successful (tailored) interventions used a minimum of 19 (weekly) contacts per patient^28^. Aranda et al. suggested that the lack of results for their intervention might be explained by the low intervention dose they used.^29^ - However, several studies of these nursing interventions had higher than expected loss to follow-up, some with greater attrition rates in the intervention group and/or among the patients having severe symptoms. This suggests that the intervention was too burdensome or too intrusive^30, 31^ - The needs assessment indicated that patients want to live as normal as possible.^9, 32-34^ Patients have expressed their wish for convenient supportive interventions that would allow them to live as normally as possible^32^. - Additionally, both panels were concerned that adopting high-dose interventions in a time of cost-savings pressure in healthcare may have little chance in getting implemented. Therefore, preference of both panels inclined towards a low-dose (standard) intervention. - Both panels believed that for a (sufficient) number of patients with cancer, a low intervention dose may be successful in achieving performance objectives. - Literature supports their experience that some patients more actively engage in self-management than others, who may need more support into doing so.^7, 8, 19^ - Regarding motivational interviewing as a strategy, brief interventions have been successful in other domains^26^. |
| **Intervention content** | First nurse counselling session | - Theory on the preparation of patients for potentially threatening medical procedures indicates the benefits of adequate and timely sensory, psychological, and procedural information, including reduced psychological distress.^35^ - Evidence suggests that unexpected symptoms cause greater distress than expected symptoms and that patients are better able to cope with expected symptoms.^36^ - A counselling session at the start of treatment is a standard part of many interventions aimed at reducing chemotherapy-related symptom burden.^27-29, 37, 38^ - Both panels believed that a counselling session at this point remained an important element of the intervention. |
|  | Second nurse counselling contact, by telephone | - Both panels believed in the importance of having a second telephone contact as an opportunity to reinforce and motivate self-care strategies as the first symptoms appeared (especially since, at the first contact, patients do not yet experience any side-effects). - Organising more than one motivational encounter seems to increase the potential of motivational interviewing interventions to be effective^26^ - Our qualitative research determined that revealing and experiencing how symptoms turn out, sets the learning process of how to deal with them in motion^39^. Also, with patients being able to express how they experience their first symptoms, their motivation to engage in self-management becomes more apparent^39^, and this provides concrete clues for motivational interviewing. - Both panels believed that a telephone contact would be convenient for the second counselling session. - Telephone-based interventions are effective in a broad range of populations, and many chemotherapy-related nursing interventions have used telephone contacts^28-31, 38, 40-42^. - Automated or digital communication strategies have also been successfully used in patients with cancer^43-45^, and were therefore considered in our intervention. However, both panels believed these newer media would not be able to reach all patients. Also, automated systems do not allow individual tailoring of communication and motivational techniques to the patient’s perception and self-management profile. Therefore at this stage of development, the telephone was chosen as a more approachable medium. |
|  | Nurses’ estimate of patient risk profile | - Given the tailored dosing of the intervention, both panels were aware that the estimation of the individual patient’s self-management profile is paramount. - After literature review, a ready-to-use instrument to assist the intervention nurses in detecting patients at risk for poor self-management seemed lacking. - Nonetheless, the patient and caregiver panel resisted proposing the use of a structured checklist or instrument for the trusting, collaborative, and equal communication approach of our intervention. - Often, clinicians make this assessment subjectively^46^. However, it was suggested and approved to include some risk factors in the intervention manual to be considered in deciding on the need for further coaching. These risk factors related to the personal context of the patient (e.g., living alone or poor social support, poor understanding of information), the perceived motivation or capability to engage in self-care as well as the performed self-care behavior, and the experienced symptom burden, as self-reported by the patient and registered by the clinical nurse - To avoid tailoring further care based on their first impression, nurses treating patients with chronic diseases prefer to use subsequent consultations to make their judgment^47^. Therefore, it was suggested and approved that CHEMO-SUPPORT nurses would report any concerns of poor self-management or indications of (in)adequate self-management. This would allow the CHEMO-SUPPORT nurse, at a subsequent contact, to take this information into account when estimating the need for further intervention. |
|  | New patient brochure | - The importance of written patient information in the outpatient setting of chemotherapy is generally accepted^48^ and many chemotherapy-specific brochures were available in the clinical setting. - However, several patients in the patient and caregiver panel felt that the information provided in the brochures was insufficient to achieve the performance objectives of the intervention. They had experienced that self-care advice wasn’t presented in a way that seemed easy to perform or in a way that seemed to promise any symptom relief. - Vicarious experiences (observing others similar to oneself successfully perform an activity) is known to enhance self-efficacy.^20^ - Patients have expressed to lack opportunity to speak to other patients while others definitely preferred not to meet with other patients.^32^ - Patients in the panel confirmed that experience of fellow patients was highly relevant but that face-to-face contact with fellow patients were too confrontational for many patients. Both panels agreed on the importance of collaborating with patients to revise the written information and of including patients’ citations to endorse the self-care advice provided. |
|  | On-call or online access to the CHEMO-SUPPORT nursing team | - Patients are reluctant to report their symptoms to professional caregivers and this reluctance is explained by both personal and environmental factors.^8^ - Telephone and e-mail availability of the CHEMO-SUPPORT team were considered to be a potentially valuable and more accessible service for non-urgent concerns or questions than having patients directly call the medical or nursing ward. - Research has shown the value of offering the possibility of personal contact in addition to the general information.^49^ |

**References**

**1.** Coolbrandt A, Van den Heede K, Clemens K, et al. The Leuven questionnaire for Patient Self-care during Chemotherapy (L-PaSC): instrument development and psychometric evaluation. *Eur J Oncol Nurs.* Jun 2013;17(3):275-283.

**2.** Dodd MJ. Assessing patient self-care for side effects of cancer chemotherapy--part I. *Cancer Nurs.* Dec 1982;5(6):447-451.

**3.** Dodd MJ. Self-care for side effects in cancer chemotherapy: an assessment of nursing interventions--Part II. *Cancer Nurs.* Feb 1983;6(1):63-67.

**4.** Given CW, Given BA, Sikorskii A, et al. Deconstruction of nurse-delivered patient self-management interventions for symptom management: factors related to delivery enactment and response. *Ann Behav Med.* Aug 2010;40(1):99-113.

**5.** Coolbrandt A, Van den Heede K, Vanhove E, De Bom A, Milisen K, Wildiers H. Immediate versus delayed self-reporting of symptoms and side effects during chemotherapy: does timing matter? *Eur J Oncol Nurs.* Apr 2011;15(2):130-136.

**6.** Homsi J, Walsh D, Rivera N, et al. Symptom evaluation in palliative medicine: patient report vs systematic assessment. *Support Care Cancer.* May 2006;14(5):444-453.

**7.** Bennion AE, Molassiotis A. Qualitative research into the symptom experiences of adult cancer patients after treatments: a systematic review and meta-synthesis. *Support Care Cancer.* Jan 1998;21(1):9-25.

**8.** Coolbrandt A, Dierckx de Casterle B, Wildiers H, et al. Dealing with chemotherapy-related symptoms at home: a qualitative study in adult patients with cancer. *Eur J Cancer Care (Engl).* Mar 6 2015.

**9.** Pedersen B, Koktved DP, Nielsen LL. Living with side effects from cancer treatment--a challenge to target information. *Scand J Caring Sci.* Sep 2012;27(3):715-723.

**10.** Williams SA, Schreier AM. The effect of education in managing side effects in women receiving chemotherapy for treatment of breast cancer. *Oncol Nurs Forum.* Jan-Feb 2004;31(1):E16-23.

**11.** Atkinson TM, Ryan SJ, Bennett AV, et al. The association between clinician-based common terminology criteria for adverse events (CTCAE) and patient-reported outcomes (PRO): a systematic review. *Support Care Cancer.* Aug 2016;24(8):3669-3676.

**12.** Basch E, Deal AM, Kris MG, et al. Symptom Monitoring With Patient-Reported Outcomes During Routine Cancer Treatment: A Randomized Controlled Trial. *J Clin Oncol.* Feb 20 2016;34(6):557-565.

**13.** Basch E, Jia X, Heller G, et al. Adverse symptom event reporting by patients vs clinicians: relationships with clinical outcomes. *J Natl Cancer Inst.* Dec 2 2009;101(23):1624-1632.

**14.** Detmar SB, Muller MJ, Schornagel JH, Wever LD, Aaronson NK. Health-related quality-of-life assessments and patient-physician communication: a randomized controlled trial. *JAMA.* Dec 18 2002;288(23):3027-3034.

**15.** Howell D, Molloy S, Wilkinson K, et al. Patient-reported outcomes in routine cancer clinical practice: a scoping review of use, impact on health outcomes, and implementation factors. *Ann Oncol.* Sep 2015;26(9):1846-1858.

**16.** Velikova G, Booth L, Smith AB, et al. Measuring quality of life in routine oncology practice improves communication and patient well-being: a randomized controlled trial. *J Clin Oncol.* Feb 15 2004;22(4):714-724.

**17.** Lorig KR, Holman H. Self-management education: history, definition, outcomes, and mechanisms. *Ann Behav Med.* Aug 2003;26(1):1-7.

**18.** Richard AA, Shea K. Delineation of self-care and associated concepts. *J Nurs Scholarsh.* Sep 2011;43(3):255-264.

**19.** Kidd L, Hubbard G, O'Carroll R, Kearney N. Perceived control and involvement in self care in patients with colorectal cancer. *J Clin Nurs.* Aug 2009;18(16):2292-2300.

**20.** Hoffman AJ. Enhancing self-efficacy for optimized patient outcomes through the theory of symptom self-management. *Cancer Nurs.* Jan-Feb 2013;36(1):E16-26.

**21.** Craddock RB, Adams PF, Usui WM, Mitchell L. An intervention to increase use and effectiveness of self-care measures for breast cancer chemotherapy patients. *Cancer Nurs.* Aug 1999;22(4):312-319.

**22.** Bartholomew LP, GS; Kok, G; Gottlieb, NH; Fernandez, ME. *Planning Health Promotion Programs: An Intervention Mapping Approach, 3rd Edition*: Jossey-Bass; 2011.

**23.** Kok G, Gottlieb NH, Peters GJ, et al. A taxonomy of behaviour change methods: an Intervention Mapping approach. *Health Psychol Rev.* Sep 2015;10(3):297-312.

**24.** Burke BL, Arkowitz H, Menchola M. The efficacy of motivational interviewing: a meta-analysis of controlled clinical trials. *J Consult Clin Psychol.* Oct 2003;71(5):843-861.

**25.** Dunn C, Deroo L, Rivara FP. The use of brief interventions adapted from motivational interviewing across behavioral domains: a systematic review. *Addiction.* Dec 2001;96(12):1725-1742.

**26.** Rubak S, Sandbaek A, Lauritzen T, Christensen B. Motivational interviewing: a systematic review and meta-analysis. *Br J Gen Pract.* Apr 2005;55(513):305-312.

**27.** Coolbrandt A, Wildiers H, Aertgeerts B, et al. Characteristics and effectiveness of complex nursing interventions aimed at reducing symptom burden in adult patients treated with chemotherapy: A systematic review of randomized controlled trials. *Int J Nurs Stud.* Mar 2014;51(3):495-510.

**28.** Molassiotis A, Brearley S, Saunders M, et al. Effectiveness of a home care nursing program in the symptom management of patients with colorectal and breast cancer receiving oral chemotherapy: a randomized, controlled trial. *J Clin Oncol.* Dec 20 2009;27(36):6191-6198.

**29.** Aranda S, Jefford M, Yates P, et al. Impact of a novel nurse-led prechemotherapy education intervention (ChemoEd) on patient distress, symptom burden, and treatment-related information and support needs: results from a randomised, controlled trial. *Ann Oncol.* Jan 2012;23(1):222-231.

**30.** Given C, Given B, Rahbar M, et al. Effect of a cognitive behavioral intervention on reducing symptom severity during chemotherapy. *J Clin Oncol.* Feb 1 2004;22(3):507-516.

**31.** Sherwood P, Given BA, Given CW, et al. A cognitive behavioral intervention for symptom management in patients with advanced cancer. *Oncol Nurs Forum.* Nov 2005;32(6):1190-1198.

**32.** Ellis J, Wagland R, Tishelman C, et al. Considerations in developing and delivering a nonpharmacological intervention for symptom management in lung cancer: the views of patients and informal caregivers. *J Pain Symptom Manage.* Dec 2012;44(6):831-842.

**33.** Steel J, Geller DA, Tsung A, et al. Randomized controlled trial of a collaborative care intervention to manage cancer-related symptoms: lessons learned. *Clin Trials.* Jun 2011;8(3):298-310.

**34.** Ruland CM, Andersen T, Jeneson A, et al. Effects of an internet support system to assist cancer patients in reducing symptom distress: a randomized controlled trial. *Cancer Nurs.* Jan-Feb;36(1):6-17.

**35.** Schofield MJ, Sanson-Fisher R. How to prepare cancer patients for potentially threatening medical procedures: consensus guidelines. NSW Cancer Council Cancer Education Research Program. *J Cancer Educ.* Fall 1996;11(3):153-158.

**36.** Boehmke MM, Dickerson SS. Symptom, symptom experiences, and symptom distress encountered by women with breast cancer undergoing current treatment modalities. *Cancer Nurs.* Sep-Oct 2005;28(5):382-389.

**37.** Jahn P, Renz P, Stukenkemper J, et al. Reduction of chemotherapy-induced anorexia, nausea, and emesis through a structured nursing intervention: a cluster-randomized multicenter trial. *Support Care Cancer.* Dec 2009;17(12):1543-1552.

**38.** Williams PD, Williams K, Lafaver-Roling S, Johnson R, Williams AR. An intervention to manage patient-reported symptoms during cancer treatment. *Clin J Oncol Nurs.* Jun 2011;15(3):253-258.

**39.** Coolbrandt A, Dierckx de Casterle B, Wildiers H, et al. Dealing with chemotherapy-related symptoms at home: a qualitative study in adult patients with cancer. *Eur J Cancer Care (Engl).* Jan 2016;25(1):79-92.

**40.** Barsevick A, Beck SL, Dudley WN, et al. Efficacy of an intervention for fatigue and sleep disturbance during cancer chemotherapy. *J Pain Symptom Manage.* Aug 2010;40(2):200-216.

**41.** Doorenbos A, Given B, Given C, Verbitsky N, Cimprich B, McCorkle R. Reducing symptom limitations: a cognitive behavioral intervention randomized trial. *Psychooncology.* Jul 2005;14(7):574-584.

**42.** Given B, Given CW, McCorkle R, et al. Pain and fatigue management: results of a nursing randomized clinical trial. *Oncol Nurs Forum.* Jul 2002;29(6):949-956.

**43.** Kearney N, Kidd L, Miller M, et al. Utilising handheld computers to monitor and support patients receiving chemotherapy: results of a UK-based feasibility study. *Support Care Cancer.* Jul 2006;14(7):742-752.

**44.** Kearney N, McCann L, Norrie J, et al. Evaluation of a mobile phone-based, advanced symptom management system (ASyMS) in the management of chemotherapy-related toxicity. *Support Care Cancer.* Apr 2009;17(4):437-444.

**45.** Ruland CM, Holte HH, Roislien J, et al. Effects of a computer-supported interactive tailored patient assessment tool on patient care, symptom distress, and patients' need for symptom management support: a randomized clinical trial. *J Am Med Inform Assoc.* Jul-Aug 2010;17(4):403-410.

**46.** Lake AJ, Staiger PK. Seeking the views of health professionals on translating chronic disease self-management models into practice. *Patient Educ Couns.* Apr;79(1):62-68.

**47.** Bos-Touwen I, Dijkkamp E, Kars M, Trappenburg J, De Wit N, Schuurmans M. Potential for Self-Management in Chronic Care: Nurses' Assessments of Patients. *Nurs Res.* Jul-Aug;64(4):282-290.

**48.** Kinnane N, Thompson L. Evaluation of the addition of video-based education for patients receiving standard pre-chemotherapy education. *Eur J Cancer Care (Engl).* Jul 2008;17(4):328-339.

**49.** Ruland CM, Andersen T, Jeneson A, et al. Effects of an internet support system to assist cancer patients in reducing symptom distress: a randomized controlled trial. *Cancer Nurs.* Jan-Feb 2013;36(1):6-17.
