# Supplementary material for: Systematic development of CHEMO-SUPPORT, a nursing intervention to support adult patients with cancer in dealing with chemotherapy-related symptoms at home
Source: BMC Nurs. 2018 Jun 27;17:28. doi: 10.1186/s12912-018-0297-8 (PMC6020323; doi:10.1186/s12912-018-0297-8)
Supplement: Supplementary file 2 — File shows evidence underpinning the needs assessment. (DOCX 66 kb) [file 12912_2018_297_MOESM2_ESM.docx]

**Additional file 2. Evidence underpinning the Needs Assessment**

| **Quality of Life and Cost** | Decreased quality of life, symptom distress, limitations in functioning, psychological and existential burden^1-6^  Treatment delay, dose reduction, treatment termination^7, 8^  Health care consumption, e.g. emergency department visits, unplanned admissions^9-11^ |
| --- | --- |
| **Health** | Chemotherapy-related symptom burden (incidence, severity, distress)^2-4^ |
| **Behaviour** | **Behavior of at-risk group**  Poor/inadequate self-management of symptoms^12-16^  Poor/inadequate communication and reporting of symptoms and symptom care needs^3, 17-20^  **Behavior of the environment**  **Family caregivers**  Not always available^21^  Engagement in other caregiver roles than coaching symptom (self-)management^21^  **Professional caregivers**  Underestimation and under-detection of symptoms^22-24^  Under-treatment of symptoms^25, 26^  Suboptimal self-management support^16, 27^ |
| **Determinants** | **Determinants explaining the behavior of at-risk group (patients)**  Lack of knowledge and experience, uncertainty^20, 28-30^  Willingness to endure^16, 31^  Sense of powerlessness, assuming that nothing can be done^16, 19, 20, 27, 31, 32^  Wanting to forget about it and live as normal as possible^20, 33-35^  Barriers to and beliefs about medication^30, 36^  Barriers to communicating on symptoms with healthcare professionals^16, 19, 20, 30, 37, 38^: lack of continuity, perceived fatalism of HCP, perceived disinterest of HCP, attitude, lack of confidence, believed lack of knowledge of HCP, poor relation or contact with HCP, *unclear communication tracks, unclear trigger events or alarm signals*, …  Difficulty of managing multiple symptoms simultaneously^28^  *Feeling depressed*  *Caregiver burden*  **Determinants at the environmental level (healthcare professionals)**  Lack of knowledge^30, 39^  Belief that nothing can be done^37^  Focus on treatment/cure^30, 39^  *Lack of concern or engagement*  *Lack of time*  *Multiprofessional care and unclear roles*  *Focus on pharmacological supportive care alone* |

Items proposed by the panels are italicized. The remaining items are from the literature.

**References**

**1.** Breen SJ, Baravelli CM, Schofield PE, Jefford M, Yates PM, Aranda SK. Is symptom burden a predictor of anxiety and depression in patients with cancer about to commence chemotherapy? *Med J Aust.* Apr 06 2009;190(7 Suppl):S99-104.

**2.** Cleeland CS, Mendoza TR, Wang XS, et al. Levels of symptom burden during chemotherapy for advanced lung cancer: differences between public hospitals and a tertiary cancer center. *J Clin Oncol.* Jul 20 2011;29(21):2859-2865.

**3.** Clover K, Kelly P, Rogers K, Britton B, Carter GL. Predictors of desire for help in oncology outpatients reporting pain or distress. *Psychooncology.* Jul 2013;22(7):1611-1617.

**4.** Kirkova J, Rybicki L, Walsh D, Aktas A. Symptom prevalence in advanced cancer: age, gender, and performance status interactions. *Am J Hosp Palliat Care.* Mar 2012;29(2):139-145.

**5.** McCorkle R, Dowd M, Ercolano E, et al. Effects of a nursing intervention on quality of life outcomes in post-surgical women with gynecological cancers. *Psychooncology.* Jan 2009;18(1):62-70.

**6.** Reece JC, Chan YF, Herbert J, Gralow J, Fann JR. Course of depression, mental health service utilization and treatment preferences in women receiving chemotherapy for breast cancer. *Gen Hosp Psychiatry.* Jul-Aug 2013;35(4):376-381.

**7.** Chau I, Cunningham D. Adjuvant therapy in colon cancer--what, when and how? *Ann Oncol.* Sep 2006;17(9):1347-1359.

**8.** Walker MS, Masaquel AS, Kerr J, et al. Early treatment discontinuation and switching in first-line metastatic breast cancer: the role of patient-reported symptom burden. *Breast Cancer Res Treat.* Apr 2014;144(3):673-681.

**9.** Hassett MJ, O'Malley AJ, Pakes JR, Newhouse JP, Earle CC. Frequency and cost of chemotherapy-related serious adverse effects in a population sample of women with breast cancer. *J Natl Cancer Inst.* Aug 16 2006;98(16):1108-1117.

**10.** Lamont EB, Yu M, He Y, Saltz L, Muss H, Zaslavsky AM. Hospital-based health care use correlates with incidence of adverse events among elderly Medicare patients treated in adjuvant chemotherapy trials (Alliance 70802). *J Geriatr Oncol.* Jul 2014;5(3):230-237.

**11.** Pittman NM, Hopman WM, Mates M. Emergency room visits and hospital admission rates after curative chemotherapy for breast cancer. *J Oncol Pract.* Mar 2015;11(2):120-125.

**12.** Coolbrandt A, Van den Heede K, Clemens K, et al. The Leuven questionnaire for Patient Self-care during Chemotherapy (L-PaSC): instrument development and psychometric evaluation. *Eur J Oncol Nurs.* Jun 2013;17(3):275-283.

**13.** Dodd MJ. Assessing patient self-care for side effects of cancer chemotherapy--part I. *Cancer Nurs.* Dec 1982;5(6):447-451.

**14.** Dodd MJ. Self-care for side effects in cancer chemotherapy: an assessment of nursing interventions--Part II. *Cancer Nurs.* Feb 1983;6(1):63-67.

**15.** Given CW, Given BA, Sikorskii A, et al. Deconstruction of nurse-delivered patient self-management interventions for symptom management: factors related to delivery enactment and response. *Ann Behav Med.* Aug 2010;40(1):99-113.

**16.** Coolbrandt A, Dierckx de Casterle B, Wildiers H, et al. Dealing with chemotherapy-related symptoms at home: a qualitative study in adult patients with cancer. *Eur J Cancer Care (Engl).* Mar 6 2015.

**17.** Coolbrandt A, Van den Heede K, Vanhove E, De Bom A, Milisen K, Wildiers H. Immediate versus delayed self-reporting of symptoms and side effects during chemotherapy: does timing matter? *Eur J Oncol Nurs.* Apr 2011;15(2):130-136.

**18.** Homsi J, Walsh D, Rivera N, et al. Symptom evaluation in palliative medicine: patient report vs systematic assessment. *Support Care Cancer.* May 2006;14(5):444-453.

**19.** Passik SD, Kirsh KL, Donaghy K, et al. Patient-related barriers to fatigue communication: initial validation of the fatigue management barriers questionnaire. *J Pain Symptom Manage.* Nov 2002;24(5):481-493.

**20.** Pedersen B, Koktved DP, Nielsen LL. Living with side effects from cancer treatment--a challenge to target information. *Scand J Caring Sci.* Sep 2012;27(3):715-723.

**21.** Ream E, Pedersen VH, Oakley C, Richardson A, Taylor C, Verity R. Informal carers' experiences and needs when supporting patients through chemotherapy: a mixed method study. *Eur J Cancer Care (Engl).* Nov 2013;22(6):797-806.

**22.** Atkinson TM, Ryan SJ, Bennett AV, et al. The association between clinician-based common terminology criteria for adverse events (CTCAE) and patient-reported outcomes (PRO): a systematic review. *Support Care Cancer.* Aug 2016;24(8):3669-3676.

**23.** Basch E, Iasonos A, McDonough T, et al. Patient versus clinician symptom reporting using the National Cancer Institute Common Terminology Criteria for Adverse Events: results of a questionnaire-based study. *Lancet Oncol.* Nov 2006;7(11):903-909.

**24.** Basch E, Jia X, Heller G, et al. Adverse symptom event reporting by patients vs clinicians: relationships with clinical outcomes. *J Natl Cancer Inst.* Dec 2 2009;101(23):1624-1632.

**25.** Henry DH, Viswanathan HN, Elkin EP, Traina S, Wade S, Cella D. Symptoms and treatment burden associated with cancer treatment: results from a cross-sectional national survey in the U.S. *Support Care Cancer.* Jul 2008;16(7):791-801.

**26.** Johnsen AT, Petersen MA, Pedersen L, Houmann LJ, Groenvold M. Do advanced cancer patients in Denmark receive the help they need? A nationally representative survey of the need related to 12 frequent symptoms/problems. *Psychooncology.* Aug 2013;22(8):1724-1730.

**27.** Bennion AE, Molassiotis A. Qualitative research into the symptom experiences of adult cancer patients after treatments: a systematic review and meta-synthesis. *Support Care Cancer.* Jan 1998;21(1):9-25.

**28.** Schumacher KL, Koresawa S, West C, et al. Putting cancer pain management regimens into practice at home. *J Pain Symptom Manage.* May 2002;23(5):369-382.

**29.** Sun V, Borneman T, Koczywas M, et al. Quality of life and barriers to symptom management in colon cancer. *Eur J Oncol Nurs.* Jul 2012;16(3):276-280.

**30.** Sun VC, Borneman T, Ferrell B, Piper B, Koczywas M, Choi K. Overcoming barriers to cancer pain management: an institutional change model. *J Pain Symptom Manage.* Oct 2007;34(4):359-369.

**31.** Spichiger E, Rieder E, Muller-Frohlich C, Kesselring A. Fatigue in patients undergoing chemotherapy, their self-care and the role of health professionals: a qualitative study. *Eur J Oncol Nurs.* Apr 2012;16(2):165-171.

**32.** Kidd L, Hubbard G, O'Carroll R, Kearney N. Perceived control and involvement in self care in patients with colorectal cancer. *J Clin Nurs.* Aug 2009;18(16):2292-2300.

**33.** Ellis J, Wagland R, Tishelman C, et al. Considerations in developing and delivering a nonpharmacological intervention for symptom management in lung cancer: the views of patients and informal caregivers. *J Pain Symptom Manage.* Dec 2012;44(6):831-842.

**34.** Ruland CM, Andersen T, Jeneson A, et al. Effects of an internet support system to assist cancer patients in reducing symptom distress: a randomized controlled trial. *Cancer Nurs.* Jan-Feb 2013;36(1):6-17.

**35.** Steel J, Geller DA, Tsung A, et al. Randomized controlled trial of a collaborative care intervention to manage cancer-related symptoms: lessons learned. *Clin Trials.* Jun 2011;8(3):298-310.

**36.** Luckett T, Davidson PM, Green A, Boyle F, Stubbs J, Lovell M. Assessment and management of adult cancer pain: a systematic review and synthesis of recent qualitative studies aimed at developing insights for managing barriers and optimizing facilitators within a comprehensive framework of patient care. *J Pain Symptom Manage.* Aug 2013;46(2):229-253.

**37.** Maguire R, McCann L, Miller M, Kearney N. Nurse's perceptions and experiences of using of a mobile-phone-based Advanced Symptom Management System (ASyMS) to monitor and manage chemotherapy-related toxicity. *Eur J Oncol Nurs.* Sep 2008;12(4):380-386.

**38.** Yates PM, Edwards HE, Nash RE, et al. Barriers to effective cancer pain management: a survey of hospitalized cancer patients in Australia. *J Pain Symptom Manage.* May 2002;23(5):393-405.

**39.** Jakobsson S, Ekman T, Ahlberg K. Components that influence assessment and management of cancer-related symptoms: an interdisciplinary perspective. *Oncol Nurs Forum.* Jul 2008;35(4):691-698.
